# Comparison of Tumor Microenvironments between Primary Tumors and Lymph Node Metastases in Head and Neck Squamous Cell Carcinoma and Their Predictive Role in Immune Checkpoint Inhibitor Treatment

**DOI:** 10.3390/cells13181557

**Published:** 2024-09-16

**Authors:** Dong Hyun Kim, Jong Seok Ahn, Mingu Kang, Gahee Park, Yoojoo Lim, Soohyun Hwang, Chan-Young Ock, Jiwon Koh, Eun-Jae Chung, Seong-Keun Kwon, Yoon Kyung Jeon, Kyeong Cheon Jung, Soon-Hyun Ahn, Bhumsuk Keam

**Affiliations:** 1 Department of Internal Medicine, Seoul National University Hospital, Seoul 03080, Republic of Korea; judekim@snu.ac.kr; 2 Department of Translational Medicine, Seoul National University College of Medicine, Seoul 03080, Republic of Korea; 3 Lunit Inc., Seoul 06241, Republic of Korea; drjsahn@gmail.com (J.S.A.); jeffkang@lunit.io (M.K.); gpark@lunit.io (G.P.); yoojoolim@lunit.io (Y.L.); soohyun.hwang@lunit.io (S.H.); ock.chanyoung@lunit.io (C.-Y.O.); 4 Department of Pathology, Seoul National University Hospital, Seoul 03080, Republic of Koreaykjeon@snu.ac.kr (Y.K.J.); jungkc66@snu.ac.kr (K.C.J.); 5 Cancer Research Institute, Seoul National University College of Medicine, Seoul 03080, Republic of Korea; 6 Department of Otorhinolaryngology-Head and Neck Surgery, Seoul National University Hospital, Seoul 03080, Republic of Korea; voicechung@snu.ac.kr (E.-J.C.); ent02@snu.ac.kr (S.-K.K.); ahnsh30@snu.ac.kr (S.-H.A.)

**Keywords:** head and neck squamous cell carcinoma, lymph node metastasis, tumor microenvironment, tumor-infiltrating lymphocyte, immune phenotype

## Abstract

The relationship between tumor microenvironments (TMEs) of regional lymph node metastases (LNMs) and primary tumors in head and neck squamous cell carcinoma (HNSCC) remains unclear. This study compared tumor-infiltrating lymphocytes (TILs) and the immune phenotype (IP), characterized by spatial TIL distribution, between primary tumors and LNMs. Twenty-one HNSCC patients with regional LNM who received immune checkpoint inhibitors (ICIs) were included. A paired comparative analysis of TIL densities and IP between primary tumors and LNMs revealed no significant difference or correlation between TIL densities in primary tumors and LNMs. Their IPs were discordant in 12 patients (57.1%). Patients with high intratumoral TIL exhibited longer progression-free survival (PFS) than those with low intratumoral TIL in both primary tumors (median, 5.2 vs. 1.3 months, *p* = 0.003) and LNMs (median, 30.2 vs. 1.3 months, *p* = 0.012). Patients with inflamed IP exhibited longer PFS than those with non-inflamed IP in both primary tumors (median, 4.5 vs. 1.3 months, *p* = 0.043) and LNMs (median, 4.1 vs. 1.3 months, *p* = 0.037). Given the lack of correlation in TIL densities, the discrepancies in IP, and the predictive value of both TMEs, evaluating the TMEs of both primary tumors and LNMs may be beneficial for the precise use of ICIs in HNSCC. There was a significant discordance between the TME of primary tumors and LNMs, with implications in survival outcomes. Therefore, evaluating the TME of both the primary tumor and LNM could be beneficial for the precise use of ICIs in HNSCC.

## 1. Introduction

Head and neck squamous cell carcinoma (HNSCC) is potentially curable with surgery or concurrent chemoradiotherapy [1]. In cases where recurrence poses curative challenges, chemotherapy is necessary. Recently, immune checkpoint inhibitors (ICIs) have been demonstrated to be effective in such cases [2,3,4,5,6]. Nevertheless, the varying efficacy of ICIs, often resulting in low response rates or hyperprogressive disease [7,8], necessitates the careful selection of patients who respond well to ICIs.

Programmed cell death ligand-1 (PD-L1) expression is used as a predictive biomarker for ICIs; however, its spatial heterogeneity and use of different scoring systems limit the applicability of this technique [9,10]. Tumor-infiltrating lymphocytes (TILs), which are vital components of the tumor microenvironment (TME), have shown promise in predicting the ICI response [10,11]. In addition, the immune phenotype (IP), which reflects the spatial distribution of TILs, may serve as a valuable predictive biomarker for the ICI response [12,13]. Furthermore, when measured using Lunit SCOPE IO, an artificial intelligence (AI)-powered spatial TIL analyzer, their potential as predictive biomarkers for ICIs across various cancer types has been reported [14,15,16]. It is hence plausible to extend their applicability to predict the outcomes of ICIs in HNSCC. However, the optimal tissue type for performing this analysis in HNSCC remains to be determined.

Regional lymph node metastasis (LNM) is clinically significant in HNSCC, and most HNSCC specimens are obtained from primary masses or LNMs [17,18]. As potential discordance has been reported between the TMEs of the primary mass and LNM for other cancers [19,20], the choice of tissue for predicting the ICI response in HNSCC must be examined. Thus, identification of the disparities between the TMEs of primary tumors and LNMs can aid in optimal personalized ICI treatment in patients with HNSCC. This study aimed to compare the TILs and IPs of primary tumors and LNMs in HNSCC, measured using Lunit SCOPE IO. We also aimed to investigate which marker is better for predicting the response to ICI treatment in patients with HNSCC.

## 2. Materials and Methods

### 2.1. Patients and Study Design

Patients with histologically confirmed recurrent and/or metastatic (R/M) HNSCC treated with ICIs between June 2014 and June 2023 were recruited. Patients who underwent surgical resection of the primary site before the use of ICIs and had regional LNM confirmed on surgical specimens were included. All patients received one of the following ICI treatments: PD-1 inhibitor monotherapy, PD-L1 inhibitor monotherapy, or a combination of PD-1/PD-L1 inhibitor with cytotoxic T-lymphocyte-associated protein 4 (CTLA-4) inhibitor. Additionally, hematoxylin and eosin (H&E)-stained archival tumor tissues were examined for TILs and IPs using Lunit SCOPE IO. Two pathologists (JK and YKJ) reviewed the histopathological slides to confirm the diagnosis. For each patient, one representative slide from the primary mass and one from the regional LNM were selected as the most appropriate slides for analysis. Patients with nasopharyngeal carcinoma were excluded from this study.

First, we compared the TMEs of the primary masses and LNMs. The variations and correlations between the TIL densities and IPs of the primary masses and LNMs were analyzed. Next, we assessed the association between ICI outcomes and the TMEs of both the primary masses and LNMs. Human papillomavirus testing, which is not mandatory for patients with oropharyngeal cancer, was conducted using p16 immunohistochemistry. The assessment of PD-L1 expression was not mandatory and was conducted using the standardized 22C3 pharmDx assay on the Dako Link 48 platform (Dako, Carpinteria, CA, USA), with the evaluation based on the combined positive score (CPS). CPS was defined as the ratio of the number of positive tumor cells, macrophages, and lymphocytes to the total number of viable tumor cells multiplied by 100 [18]. Tumor responses were assessed based on RECIST version 1.1 and were categorized as complete response (CR), partial response (PR), stable disease (SD), and progressive disease (PD) [21].

### 2.2. Procedures

Lunit SCOPE IO (Lunit Inc., Seoul, Republic of Korea) is an AI-powered spatial TIL analyzer. It identifies and quantifies TILs within the cancer epithelium (intratumoral TILs; iTIL) and stroma (stromal TILs; sTIL) from H&E-stained whole-slide images (WSIs). It employs two convolutional neural networks; one performs segmentation of the tumor region and cancer-related stroma, whereas the other identifies TILs. Lunit SCOPE IO was originally trained and optimized using 2.8 × 10^9^ μm^2^ H&E-stained tissue regions containing 6.0 × 10^5^ TILs extracted from 3166 WSIs assorted from 25 different tumor types and annotated by board-certified pathologists [16]. The model used in this study was further trained using 1.4 × 10^10^ μm^2^ of tumor tissue and stroma, containing 6.23 × 10^5^ TILs, extracted from 18,679 H&E-stained WSIs of 17 different solid tumor types. Specifically, 1462 WSIs of lymph node tissue were used for training and validating the model.

TIL analysis using Lunit SCOPE IO has been detailed in our previous study [22]. Briefly, the model estimates the density (quantity/mm^2^) of iTILs or sTILs. The model then assesses the IP of 0.25 mm^2^ grids based on their TIL density as follows: iTIL density ≥ 130/mm^2^, inflamed grids; iTIL density < 130/mm^2^ and sTIL density ≥ 260/mm^2^, immune-excluded grids; and iTIL density < 130/mm^2^ and sTIL density < 260/mm^2^, immune-desert grids. The inflamed score (IS), immune-excluded score (IES), and immune-desert score (IDS) of the WSIs were calculated by dividing the number of annotated grids for a specific IP by the total number of analyzed grids. The representative IP of each WSI was then assessed as follows: inflamed IP when IS ≥ 20.0%, immune-excluded IP when IES ≥ 33.3% and IS < 20.0%, and immune-desert IP otherwise. The TIL and immune score threshold values used for IP classification were predetermined based on their optimal ability to predict high interferon-γ-responsive gene signature levels in The Cancer Genome Atlas pan-carcinoma tumor samples (N = 7454) [23,24].

### 2.3. Statistical Analysis

Categorical variables between the two groups were compared using Fisher’s exact test or the chi-square test, and *p*-values were estimated using two-sided tests. Differences in the means or medians of the continuous variables between the two groups were assessed using the Wilcoxon rank-sum test. For paired data, the paired *t*-test or Wilcoxon signed-rank was used for continuous variables, whereas the Stuart-Maxwell test was used for categorical variables. Pearson’s correlation coefficient was used to assess correlations between two variables. The cut-off for discriminating between high and low TIL densities was defined as the point with the highest log-rank statistics for progression-free survival (PFS), as determined by the maximally selected rank statistics. The Kaplan–Meier method was used to estimate the PFS. The log-rank test was used to assess the differences between the groups in terms of the PFS. The statistical software ‘R’ version 4.3.1 (www.r-project.org (accessed on 1 September 2023)) was used for all statistical analyses. *p*-values of <0.05 were considered statistically significant.

## 3. Results

### 3.1. Patient Characteristics

Samples from 22 patients were identified and analyzed using Lunit SCOPE IO. The sample from one patient did not pass the Lunit SCOPE IO quality control checks owing to an insufficient grid count for the TIL density calculation. Consequently, samples from 21 patients were analyzed. The baseline characteristics of these patients are presented in Table 1. The median age was 55 (range, 36–72) years, and 16 (76.2%) patients were male. At the initiation of ICI treatment, lung and bone metastases were present in 14 (66.7%) and 4 (19%) patients, respectively. PD-L1 CPS was less than 1 in one out of nine evaluable patients.

### 3.2. Comparison of the TMEs of Primary Tumors and LNMs

The Lunit SCOPE IO analysis results are summarized in Table 2. In primary tumors and LNMs, the median iTIL densities were 57.71/mm^2^ (range, 3.32–753.19) and 105.29/mm^2^ (range, 4.01–1559.75), respectively, whereas the median sTIL densities were 1153.21/mm^2^ (range, 160.45–8725.75) and 1868.17/mm^2^ (range, 78.1–14,728.41), respectively. The IP of each grid was determined for the spatial analysis of TILs. In the primary tumors, the median IS, IES, and IDS values were 13.4%, 29.9%, and 41.6%, respectively, whereas, in LNMs, these values were 20.5%, 33%, and 33.4%, respectively. There were no statistically significant differences between the values of iTIL density, sTIL density, IS, IES, and IDS of the primary tumors and LNMs, with *p*-values of 0.424, 0.237, 0.837, 0.781, and 0.628, respectively (Figure S1).

The densities of iTIL and sTIL were strongly associated with each other in both primary tumors (r = 0.809, *p* < 0.001) and LNMs (r = 0.823, *p* < 0.001). However, the iTIL density in the primary tumors was not significantly correlated with that in LNMs (r = 0.386, *p* = 0.084); a similar trend was observed for sTIL density as well (r = 0.239, *p* = 0.297) (Figure S2).

**Figure 1:**
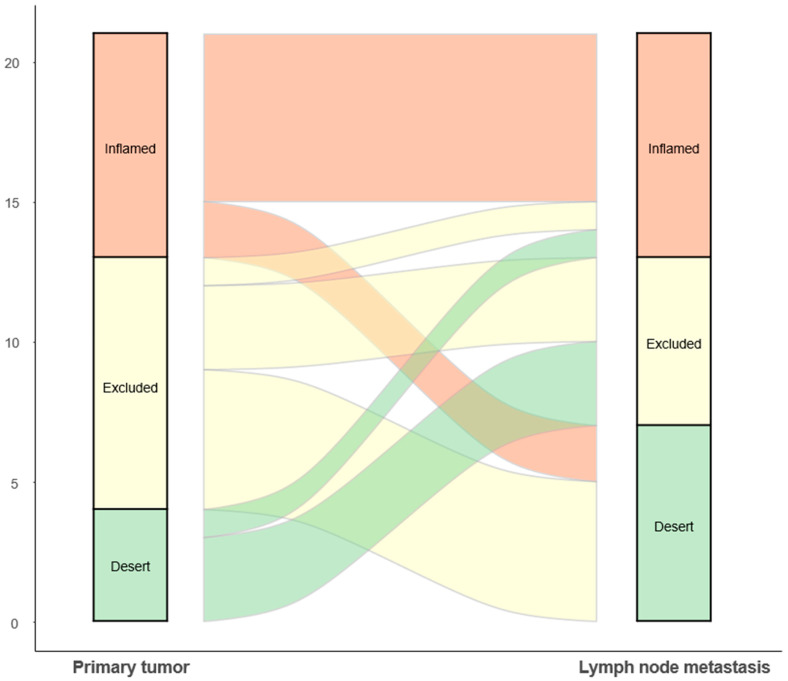
Sankey plot showing changes in the immune phenotype.

**Figure 2:**
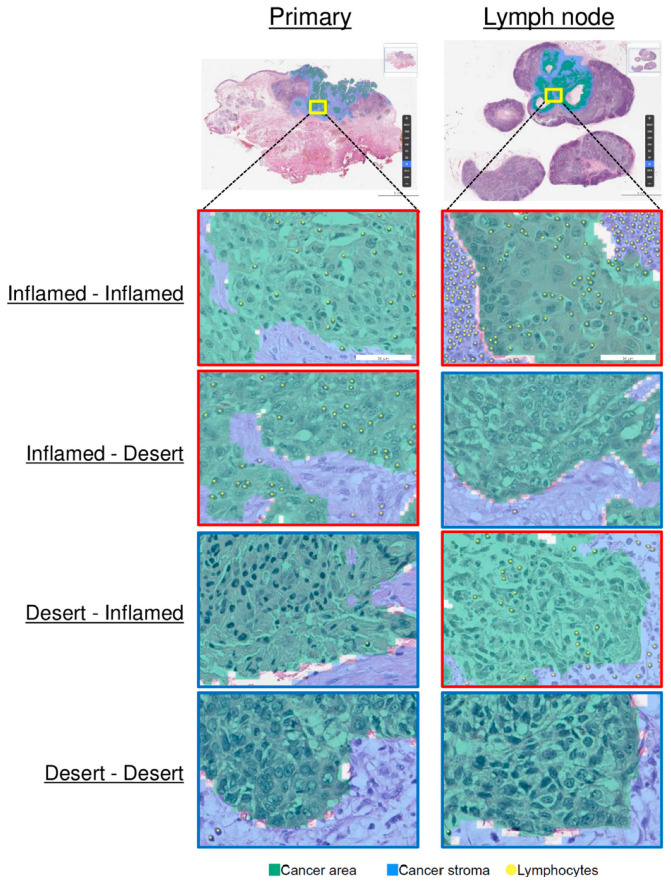
Representative image of cancerous areas using Lunit SCOPE IO. Lunit SCOPE IO-inferenced images from four patients where the immune phenotypes of the primary tumor and lymph node metastasis are inflamed-inflamed, inflamed-desert, desert-inflamed, and desert-desert, respectively.

The distribution of IPs showed no significant difference between the primary tumors and LNMs (*p* = 0.598); in the primary tumors, eight were inflamed (38.1%), nine were immune-excluded (42.9%), and four were desert (19.0%); in the LNMs, eight were inflamed (38.1%), six were immune-excluded (28.6%), and seven were desert (33.3%). Among the 21 patients, the IP of the primary tumor matched that of the LNM in 9 (42.9%) patients, while, in the remaining 12 (57.1%) patients, the IP of the primary tumor differed from that of the LNM (Figure 1). Specifically, all four patients with a desert IP in the primary tumor exhibited inflamed or immune-excluded IP in the LNM. None of the seven patients with a desert IP in the LNM had a desert IP in the primary tumor. Thus, the observation of a desert IP in the primary tumor did not correspond to a desert IP in the LNM. In 10 (47.6%) patients, the IP was concordantly non-desert in both the primary tumor and LNM. In four (19%) patients, the primary tumor was characterized by a desert IP while the LNM was non-desert, and the opposite was observed in seven (33.3%) patients (Figure 2).

### 3.3. Relationship between ICI Efficacy and the TME

The best responses to ICI were CR for 2 (9.5%), PR for 2 (9.5%), SD for 3 (14.3%), and PD for 14 (66.7%) patients (Table S1). The overall response rate was 19.0% (95% confidence interval [CI], 5.4–41.9%), and the disease control rate was 33.3% (95% CI, 14.6–57.0%). With a median follow-up period of 21.7 months, the median PFS was 1.7 months (95% CI, 1.3–5.2 months).

**Figure 3:**
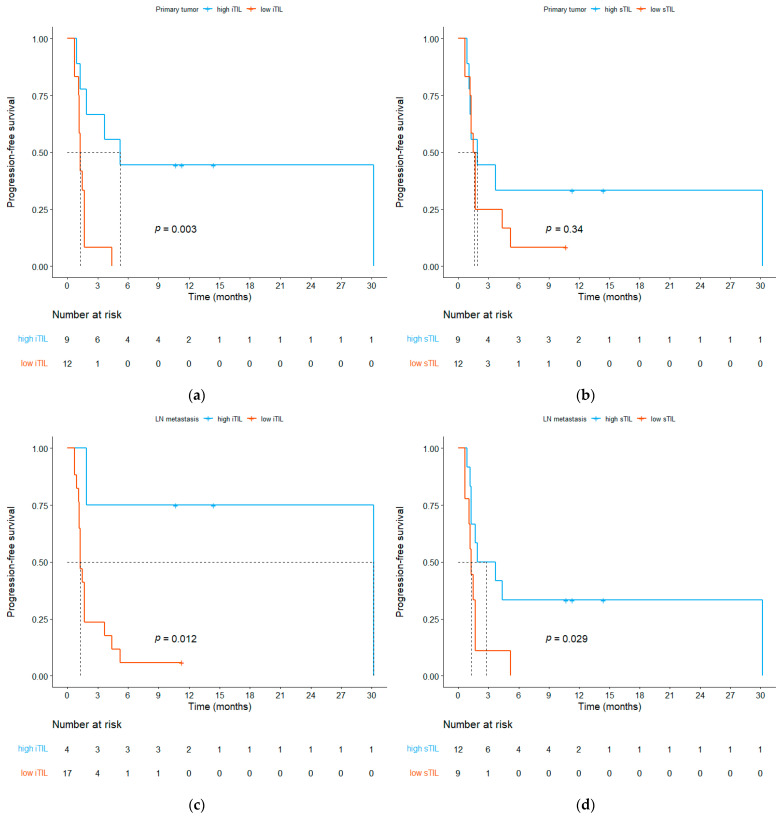
Kaplan–Meier curves of progression-free survival based on tumor-infiltrating lymphocyte (TIL) density. (**a**) High intratumoral TIL (iTIL) vs. low iTIL in the primary tumor; (**b**) high stromal TIL (sTIL) vs. low sTIL in the primary tumor; (**c**) high iTIL vs. low iTIL in lymph node metastasis; (**d**) high sTIL vs. low sTIL in lymph node metastasis.

We categorized the patients into responders and non-responders based on their best responses and subsequently compared the TME between these two groups (Table 3). Responders (who exhibited CR and PR) exhibited higher iTIL densities in both the primary tumor (median, 297.9 vs. 45.17/mm^2^, *p* = 0.031) and LNM (median, 377.4 vs. 60.53/mm^2^, *p* = 0.031) than non-responders (who exhibited SD and PD). Additionally, patients with PD had lower iTIL densities in both the primary tumor (median, 41.54 vs. 275.95/mm^2^, *p* = 0.038) and LNM (median, 55.86 vs. 270.4/mm^2^, *p* = 0.02) than those with disease control (CR, PR, or SD). In contrast, no differences in sTIL density were observed with respect to the overall response or disease control in either the primary tumors or LNMs. Then, we compared the PFS between patients with high and low TIL densities. The optimal cut-off levels for determining the high and low iTIL density in the primary tumors and LNM were 70.62 and 270.44, respectively, whereas the corresponding cut-off levels for sTIL were 1254.5 and 1179.2. For the primary tumors, patients with a high iTIL density (>70.62/mm^2^) exhibited longer PFS (Figure 3a, median 5.2 vs. 1.3 months, *p* = 0.003). However, PFS did not differ between high and low sTIL density (Figure 3b, median 1.9 vs. 1.6 months, *p* = 0.34). In contrast, for LNM, patients with high iTIL (>270.44/mm^2^) and sTIL (>1179.2/mm^2^) densities each exhibited longer PFS compared to those with low densities (Figure 3c, median 30.2 vs. 1.3 months, *p* = 0.012; Figure 3d, median 2.8 vs. 1.3 months, *p* = 0.029).

**Figure 4:**
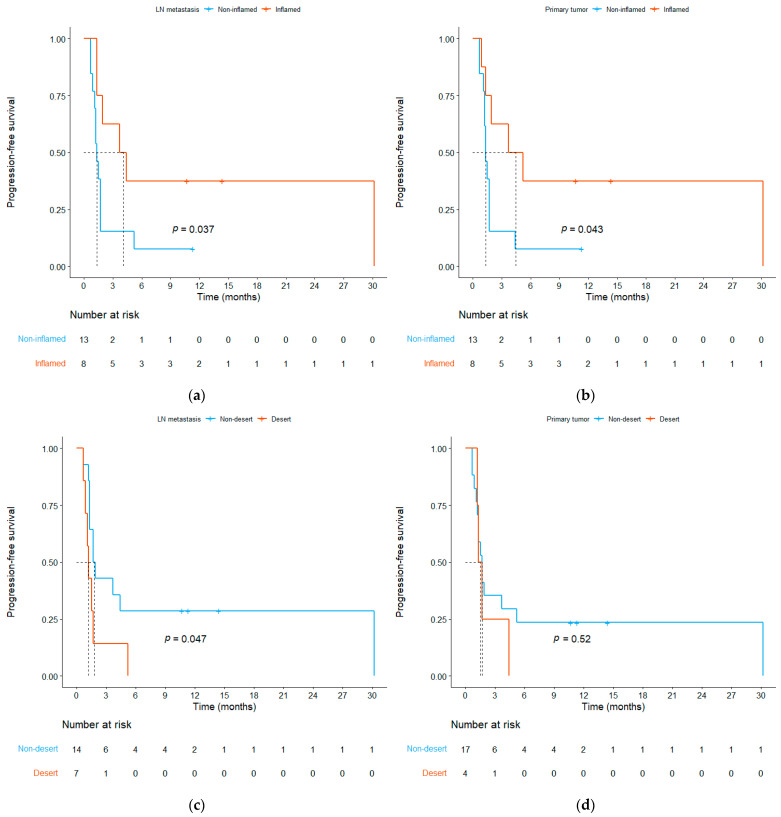

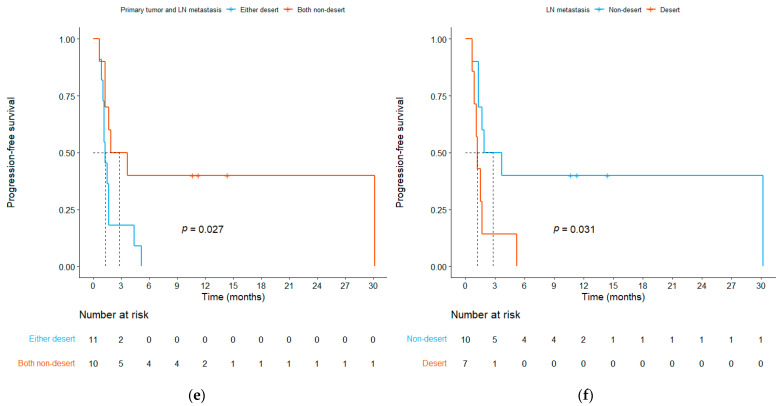
Kaplan–Meier curves of progression-free survival based on immune phenotype (IP). (**a**) Inflamed vs. non-inflamed IP in lymph node metastasis. (**b**) Inflamed vs. non-inflamed IP in the primary tumor. (**c**) Desert vs. non-desert IP in lymph node metastasis. (**d**) Desert vs. non-desert IP in the primary tumor. (**e**) Non-desert IP in both the primary tumor and lymph node metastasis vs. desert IP in either site. (**f**) Desert vs. non-desert IP in lymph node metastasis in patients with a non-desert primary tumor.

To determine whether IP is a predictive biomarker for ICI treatment, we analyzed survival outcomes according to the IP classification in both primary tumors and LNMs. Patients with inflamed IP demonstrated significantly longer PFS than those with non-inflamed IP in both the LNM (Figure 4a, median 4.1 vs. 1.3 months, *p* = 0.037) and primary tumor (Figure 4b, median 4.5 vs. 1.3 months, *p* = 0.043). On comparing desert and non-desert IPs, desert IP was associated with a shorter PFS in LNMs (Figure 4c, median 1.2 vs. 1.8 months, *p* = 0.047). However, for primary tumors, PFS did not differ between desert and non-desert IPs (Figure 4d, median 1.5 vs. 1.7 months, *p* = 0.52).

We further focused on the IP discrepancies between the primary tumor and LNM. Patients with non-desert IPs in both sites exhibited significantly longer PFS than those with desert IP in either site (Figure 4e, median 2.8 vs. 1.3 months, *p* = 0.027). This analysis yielded a more significant log-rank test result compared to when the PFS was analyzed based on the IPs of the primary tumor or LNM alone. PFS did not differ based on whether the primary tumor was a desert or non-desert IP; however, among those with non-desert primary tumors, patients with desert LNM had shorter PFS compared to those with non-desert LNM (Figure 4f, median 1.2 vs. 2.8 months, *p* = 0.031).

## 4. Discussion

The present study aimed to evaluate tumor heterogeneity between primary tumors and nodal metastasis in terms of TILs and IP. We conducted a comparative analysis of TIL density and IP between primary tumors and regional LNMs to assess the differences in their TMEs. Our findings revealed that neither iTIL nor sTIL densities in the primary tumor were correlated with those in LNMs. For more than 50% of patients, the IP of the primary tumor differed from that of LNM. With respect to the ICI treatment response, responders exhibited higher iTIL density in both the primary tumor and LNM than non-responders. High iTIL in the primary tumor and both high iTIL and sTIL in the LNM were associated with longer PFS. Inflamed IP in the primary tumor and inflamed or non-desert IP in the LNM were associated with longer PFS than their respective counterparts. Furthermore, considering the IP of both the primary tumor and LNM may provide a better prediction of ICI outcomes.

Most previous studies comparing primary tumors and LNMs in HNSCC have predominantly focused on PD-L1 expression, reporting mainly on their concordance. Chen et al. reported moderate agreement in PD-L1 positivity between primary tumors and LNMs [25]. PD-L1 concordance rates of more than 50% have been reported between primary tumors and LNMs in other studies as well [26,27]. Brcic et al. reported a high concordance rate between categorical TIL scores in primary tumors and LNMs [28]. However, these studies employed different methodologies and cut-off values and relied on categorical analyses. In a study of PD-L1 expression in HNSCC with paired LNMs by Kaur et al., the PD-L1 scores were concordant in over 80% of the cases when considered as dichotomous variables; however, as continuous variables, the PD-L1 score showed poor agreement [29]. Our study employed AI technology to precisely assess the TIL density and revealed the absence of a correlation between TIL densities in primary tumors and LNMs.

Regional LNM is clinically significant in the biology and prognosis of HNSCC [30]. The TME of a regional LNM can significantly affect the disease course, as LNM can serve as a potential source of further dissemination beyond simply reflecting cancer aggressiveness [17]. The lack of correlation in TIL densities between primary tumors and LNMs observed in our study is not surprising, given the tumor heterogeneity and consequential secondary impact on the TME [31,32]. In addition, genetic differences between primary tumors and LNMs have been reported previously in patients with HNSCC. Genetic alterations in LNMs are associated with the loss of adhesion molecules such as *CTNNB1*, emphasizing the clinical impact of LNMs in cancer trajectory [33,34]. Differential gene profiles of primary tumors and LNMs, along with the association of LNM-related gene signatures with TIL density [35], suggest that understanding these differences can aid in improving ICI treatment for HNSCC [36].

The prognostic and predictive values of TILs and IP in the use of ICIs are increasingly being reported in various cancer types, including HNSCC [37,38]. However, there is no established consensus on whether to use tissues from initial or recurrent tumors in R/M HNSCC. Previous studies have revealed that, in several cancer types, including HNSCC, the TIL levels decrease in recurrent tumors compared to the initial tumors, leading to progression toward an immune-desert phenotype [39,40,41]. Although this study had the limitation of a small sample size that precluded identification of a statistical difference, our findings suggest that LNMs may have a higher frequency of desert IPs compared to primary tumors, indicating a potential role of LNMs as precursors to systemic metastases [42]. Our study showed that TMEs often differ between primary tumors and LNMs; specifically, in 19% of the patients, desert IP in the primary tumor turned into non-desert IP in LNM. This suggests that obtaining a new biopsy for LNM and conducting an AI-powered TME analysis would be prudent, especially considering the scarce treatment options for HNSCC. Additionally, the present study demonstrated the predictive role of TMEs in both primary tumors and LNMs for ICI use, advocating TME evaluation of both the primary mass and LNM for future ICI treatments.

This study has a few limitations. First, the sample size was small. This might contribute to the lack of statistical significance of our findings. We were also unable to analyze the factors influencing the differences in the TME between primary tumors and LNMs; therefore, large-scale studies are required to further explore the clinical significance of the discordance in the TME. Second, there is a lack of data on PD-L1 expression, which prevented the analysis of the relationship between PD-L1 and TIL densities. Although PD-L1 expression has been compared between primary tumors and LNMs in several studies, analyzing PD-L1 in conjunction with changes in the TIL density provides a deeper understanding of its impact on ICI efficacy in the TME.

Despite these limitations, to the best of our knowledge, our study is the first to compare TMEs in terms of TIL density and IP between primary tumors and regional LNMs in HNSCC. Furthermore, we employed AI to detect TILs and define the IP of each sample. Given the challenges in defining the tumor margin and stromal area in LNM [28], we used Lunit SCOPE IO, a validated AI-powered TIL analyzer that provides a standardized method for a sample evaluation, which suggested the possibility of discrepancies in IP between the primary tumor and LNM.

## 5. Conclusions

In conclusion, this study demonstrated that the TIL density between the primary tumor and regional LNM is unrelated, and there may be frequent discrepancies in IP. Although TILs and IPs in both the primary tumor and LNM individually have predictive value for response to ICI treatment, considering both together can enhance the prediction accuracy of ICI treatment outcomes. Therefore, once LNM is confirmed during the surgical resection of the primary mass, assessing the TME in both the primary tumor and LNM can offer valuable insights into ICI treatment strategies for HNSCC.

## Figures and Tables

**Table 1:** Baseline characteristics.

Variables	N = 21
Median age, years (range)	55 (36–72)
Age > 60, n (%)	6 (28.6)
Sex, n (%)	
Male	16 (76.2)
Female	5 (23.8)
Current or former smokers, n (%)	8 (38.1)
Primary tumor location, n (%)	
Oral cavity	11 (52.4)
Oropharynx	8 (38.1)
Hypopharynx	2 (9.5)
HPV p16 status, n (%)	
Positive	7 (33.3)
Negative	5 (23.8)
Missing	9 (42.9)
PD-L1 expression, n (%)	
CPS < 1	1 (4.8)
CPS ≥ 1	8 (38.1)
Not assessed	12 (57.1)
Number of previous lines of chemotherapy before ICI, n (%)	
0	6 (28.6)
1	6 (28.6)
2 or more	9 (42.9)
Immune checkpoint inhibitor treatment, n (%)	
Pembrolizumab	11 (52.4)
Nivolumab	5 (23.8)
Nivolumab + Ipilimumab	1 (4.8)
Durvalumab	3 (14.3)
Durvalumab + Tremelimumab	1 (4.8)

HPV, human papillomavirus; PD-L1, programmed cell death ligand-1; CPS, combined positive score; ICI, immune checkpoint inhibitor.

**Table 2:** Comparison of the TMEs of the primary tumors and LNMs.

	Primary Tumor N = 21	LNM N = 21	*p*-Value
Median TIL density, /mm^2^ (range)			
Intratumoral	57.71 (3.32–753.19)	105.29 (4.01–1559.75)	0.424
Stromal	1153.2 (160.4–8725.8)	1868.2 (78.1–14,728.4)	0.237
Immune score, %, median (range)			
Inflamed score	13.41 (0.47–73.95)	20.48 (0–89.09)	0.837
Immune-excluded score	29.86 (12.85–55.33)	33.01 (4.49–68.25)	0.781
Immune-desert score	41.64 (0.84–84.5)	33.43 (2.91–95.06)	0.628
Immune phenotype, n (%)			0.598
Inflamed	8 (38.1)	8 (38.1)	
Immune-excluded	9 (42.9)	6 (28.6)	
Desert	4 (19.0)	7 (33.3)	

LNM, lymph node metastasis; TILs, tumor-infiltrating lymphocytes.

**Table 3:** Comparison of the TME based on the ICI response.

	Overall Response		*p*-Value
	CR + PR (N = 4)	SD + PD (N = 17)	
Median iTIL density, /mm^2^ (range)			
Primary tumor	297.9 (74.9–370.4)	45.17 (3.32–753.19)	0.031
Lymph node metastasis	377.4 (87.94–1559.8)	60.53 (4.01–1490.14)	0.031
Median sTIL density, /mm^2^ (range)			
Primary tumor	1328.2 (428.9–5850.4)	883.6 (160.4–8725.8)	0.574
Lymph node metastasis	3495.6 (1397.6–14,728.4)	1179.2 (78.1–7357.2)	0.144
Immune phenotype, n (%)			
Primary tumor			0.215
Inflamed	3 (75)	5 (29.4)	
Immune-excluded	1 (25)	8 (47.1)	
Desert	0 (0)	4 (23.5)	
Lymph node metastasis			0.179
Inflamed	3 (75)	5 (29.4)	
Immune-excluded	1 (25)	5 (29.4)	
Desert	0 (0)	7 (41.2)	
	**Disease Control**		***p*** **-Value**
	**CR + PR + SD** **(N = 7)**	**PD** **(N = 14)**	
Median iTIL density, /mm^2^ (range)			
Primary tumor	275.95 (11.06–753.19)	41.54 (3.32–322.17)	0.038
Lymph node metastasis	270.4 (52.95–1559.75)	55.86 (4.01–1490.14)	0.020
Median sTIL density, /mm^2^ (range)			
Primary tumor	1254.7 (160.4–8725.8)	1018.4 (281.5–3773.1)	0.971
Lymph node metastasis	3112.6 (93.9–14,728.4)	1103.7 (78.1–7357.2)	0.197
Immune phenotype, n (%)			
Primary tumor			0.075
Inflamed	5 (71.4)	3 (21.4)	
Immune-excluded	1 (14.3)	8 (57.1)	
Desert	1 (14.3)	3 (21.4)	
Lymph node metastasis			0.084
Inflamed	5 (71.4)	3 (21.4)	
Immune-excluded	1 (14.3)	5 (35.7)	
Desert	1 (14.3)	6 (42.9)	

CR, complete response; PR, partial response; SD, stable disease; PD, progressive disease; iTIL, intratumoral tumor-infiltrating lymphocytes; sTIL, stromal tumor-infiltrating lymphocytes.

## Data Availability

The datasets generated during the current study are available from the corresponding author upon reasonable request.

## Supplementary Materials

The following supporting information can be downloaded at https://www.mdpi.com/article/10.3390/cells13181557/s1: Figure S1: Comparison of the TME between the primary tumors and LNMs; Figure S2: Correlation of the TIL density between the primary tumors and LNMs; Table S1: Clinical activity of the immune checkpoint inhibitor.
