# Supplementary material for: Comparison of Tumor Microenvironments between Primary Tumors and Lymph Node Metastases in Head and Neck Squamous Cell Carcinoma and Their Predictive Role in Immune Checkpoint Inhibitor Treatment
Source: Cells. 2024 Sep 16;13(18):1557. doi: 10.3390/cells13181557 (PMC11429639; doi:10.3390/cells13181557)
Supplement: Supplementary file 1 [file cells-13-01557-s001.zip › cells-3192130-supplementary.pdf]

Supplemental Materials

Figure S1. Comparison of tumor microenvironment between primary tumors and lymph node metastases. (A) Intratumoral TIL; (B) Stromal TIL; (C) Immune phenotype score.

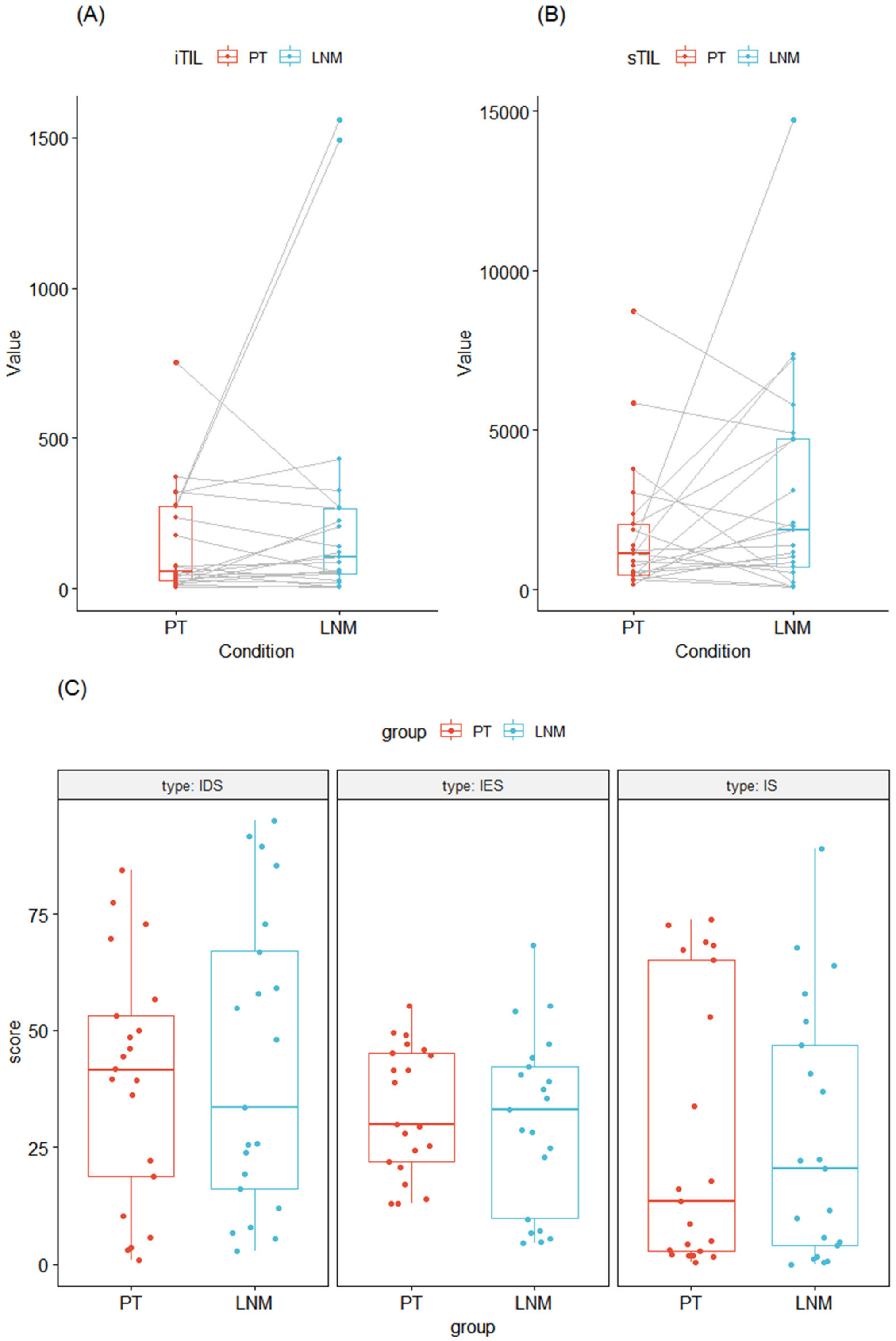

Figure S2. Correlation of TIL density between primary tumors and lymph node metastases.

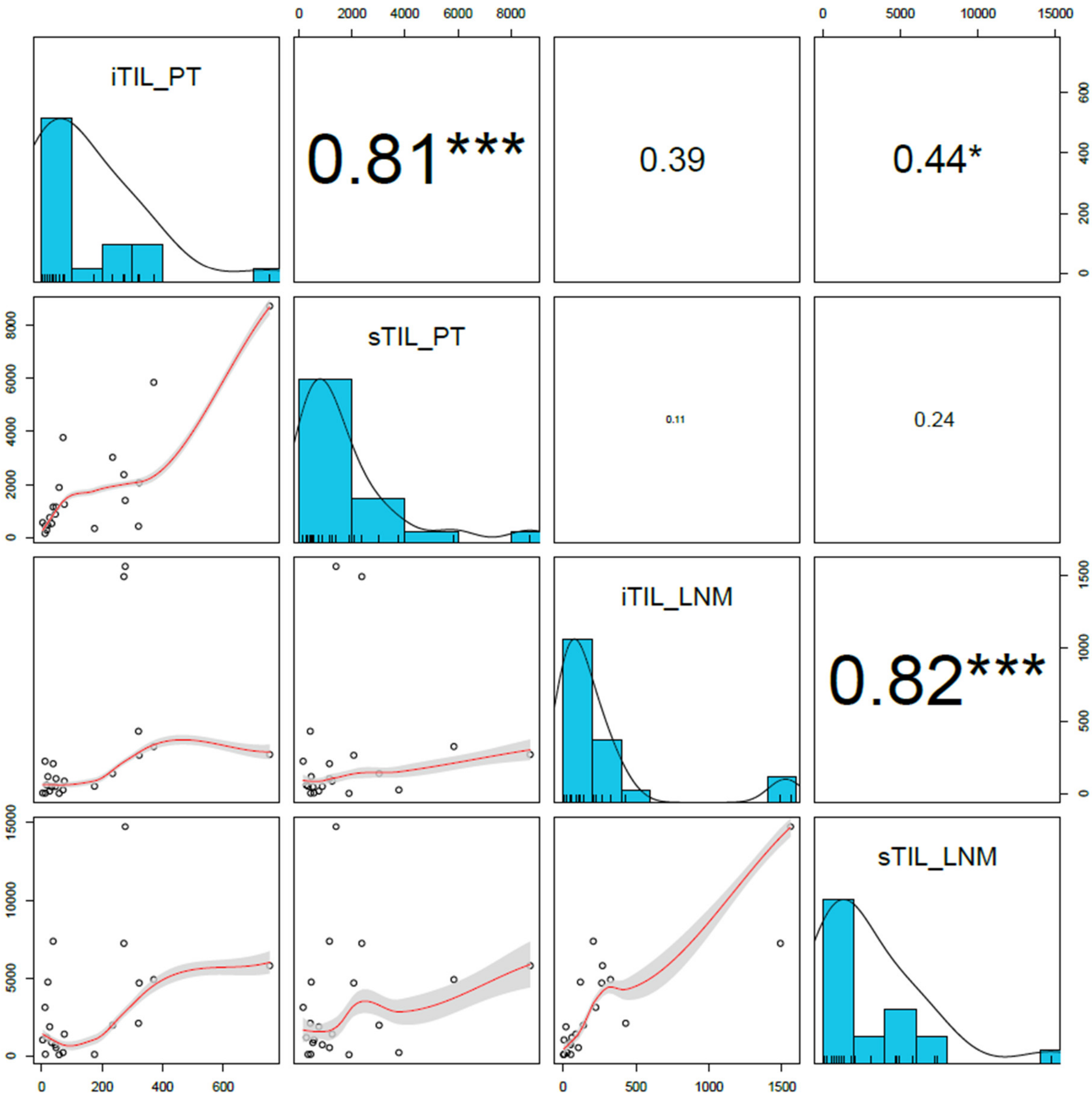

The upper half of the diagonal represents correlation coefficients, with larger font sizes indicating strong correlations, and asterisks denote statistical significance. The bottom half displays a correlation plot between the two variables.

Abbreviation: TIL, tumor-infiltrating lymphocyte; iTIL, intratumoral TIL; sTIL, stromal TIL; PT, primary tumor; LNM, lymph node metastasis.

Table S1. Clinical activity of immune checkpoint inhibitor.

	**N = 21**
**Best response, n (%)**	
Complete response	2 (9.5)
Partial response	2 (9.5)
Stable disease	3 (14.3)
Progressive disease	14 (66.7)
**Overall response rate, % (95% CI)**	19.0 (5.4 – 41.9)
**Disease control rate, % (95% CI)**	33.3 (14.6 – 57)
**PFS, months, median (95% CI)**	1.7 (1.3 – 5.2)
3 months PFS rate, % (95% CI)	33.3 (18.2 – 61.0)
6 months PFS rate, % (95% CI)	19.0 (7.9 – 46.0)

CI, confidence interval; PFS, progression-free survival.
